# Acute Late-Stage Myocarditis in the Crab-Eating Macaque Model of Hemorrhagic Smallpox

**DOI:** 10.3390/v13081571

**Published:** 2021-08-09

**Authors:** Reed F. Johnson, Lauren A. Keith, Timothy K. Cooper, Srikanth Yellayi, Nicole M. Josleyn, Krisztina B. Janosko, James D. Pettitt, David Thomasson, Katie R. Hagen, Robin Gross, John G. Bernbaum, Debbie Douglas, Jeffrey Solomon, Mark Martinez, Kurt Cooper, Marisa St. Claire, Danny R. Ragland, Peter B. Jahrling, Jens H. Kuhn, Andrew E. Arai

**Affiliations:** 1 Emerging Viral Pathogens Section, National Institute of Allergy and Infectious Diseases, National Institutes of Health, Fort Detrick, Frederick, MD 21702, USA; jahrlingp@nih.gov; 2 Integrated Research Facility at Fort Detrick, National Institute of Allergy and Infectious Diseases, National Institutes of Health, Frederick, MD 21702, USA; laurenashleykeith@gmail.com (L.A.K.); timothy.cooper@nih.gov (T.K.C.); sriky9068@gmail.com (S.Y.); nicole.m.josleyn.ctr@mail.mil (N.M.J.); krisztina.janosko@nih.gov (K.B.J.); james.pettitt@mappbio.com (J.D.P.); david@sanyasin.net (D.T.); katierhagen@gmail.com (K.R.H.); robin0429@icloud.com (R.G.); bernbaumjg@niaid.nih.gov (J.G.B.); deb.dougdallas@gmail.com (D.D.); mark66martinez@gmail.com (M.M.); kurt.cooper@nih.gov (K.C.); stclairem@niaid.nih.gov (M.S.C.); raglandd@niaid.nih.gov (D.R.R.); kuhnjens@niaid.nih.gov (J.H.K.); 3 Clinical Monitoring Research Program Directorate, Frederick National Laboratory for Cancer Research, Frederick, MD 21701, USA; jeffrey.solomon@nih.gov; 4 Advanced Cardiovascular Imaging Group, National Heart, Lung, and Blood Institute, National Institutes of Health, Bethesda, MD 20892, USA; andrewarai@hotmail.com

**Keywords:** cowpox, CPXV, smallpox, variola, VARV, myocarditis, cardiac MRI, biodefense

## Abstract

Hemorrhagic smallpox, caused by variola virus (VARV), was a rare but nearly 100% lethal human disease manifestation. Hemorrhagic smallpox is frequently characterized by secondary bacterial infection, coagulopathy, and myocardial and subendocardial hemorrhages. Previous experiments have demonstrated that intravenous (IV) cowpox virus (CPXV) exposure of macaques mimics human hemorrhagic smallpox. The goal of this experiment was to further understand the onset, nature, and severity of cardiac pathology and how it may contribute to disease. The findings support an acute late-stage myocarditis with lymphohistiocytic infiltrates in the CPXV model of hemorrhagic smallpox.

## 1. Introduction

Smallpox, one of the deadliest human infectious diseases in recorded history, caused by variola virus (VARV; *Poxviridae*: *Orthopoxvirus*) was declared eradicated in 1980 after a worldwide vaccination program [1]. Now, VARV stocks are maintained in only two institutes in the world, and any research involving replicative VARV requires approval from the World Health Organization and use of maximum containment laboratories [2]. However, development of medical countermeasures against smallpox continues to be a priority due to concerns about deliberate re-introduction of illegally acquired or synthesized VARV into an increasingly unvaccinated population [3].

Smallpox was classified based on clinical presentation and severity into hemorrhagic, flat, ordinary, and modified varieties with multiple subcategories [4]. Although rare, hemorrhagic smallpox occurred primarily in adults (vaccinated and unvaccinated) and had a 94–100% case fatality rate. Clinical characteristics included rapid onset, fever accompanied by a pathognomonic rash, severe headache, backache, restlessness, prostration, difficulty breathing, and pallor. Additional clinical findings were thrombocytopenia, increased coagulation time, albuminuria, hepatomegaly, and secondary bacterial infections. Heart failure and pulmonary edema caused death, typically around six days after fever onset [5]. Some patients presented with myocardial and endocardial hemorrhages [5,6,7], which were not characterized as contributions to disease severity and outcome.

Previously, we have demonstrated that intravenous (IV) exposure of crab-eating macaques (*Macaca fascicularis* Raffles, 1821) to the orthopoxvirus cowpox virus (CPXV) resulted in a lethal hemorrhagic smallpox-like disease characterized by rapid disease progression, coagulopathy, hemorrhagic lesions, a pro-inflammatory response, and bone marrow depletion. Interestingly, we observed lymphohistiocytic myocarditis with myocardiocyte degeneration and hemorrhage in some animals. CPXV antigen was observed in myocardial and inflammatory cell infiltrates but, given the experimental design and rapidity of the disease, further assessment of changes in cardiac tissue and function was not feasible [8]. Here, we report the targeted characterization of CPXV-induced cardiac lesions, including functional evaluation, in crab-eating macaques.

## 2. Materials and Methods

### 2.1. Virus and Cells

CPXV strain Brighton Red, provided by Grant McFadden at University of Florida, was propagated and titered on Vero E6 cells as previously described [8].

### 2.2. Study Design

The experiment was performed under biosafety level 4 (BSL-4) conditions due to facility layout. Twelve crab-eating (a.k.a. cynomolgus) macaques (Haplorrhini: Cercopithecidae: *Macaca fascicularis* Raffles, 1821) of Chinese origin were randomly divided into four groups. Group 1 consisted of two males and one female (age range 7–9 yr); Group 2 consisted of two males and one female (age 7 yr); Group 3 consisted of two males and one female (age range 7–10 yr); and the control group consisted of one male and two females (age range 7–10 yr). All subjects underwent at least two baseline physical examinations and at least two cardiac magnetic resonance imaging (cMRI) sessions. Group 1 macaques were euthanized on Day 3 post-exposure, Group 2 macaques were euthanized on Day 6 post-exposure, and Group 3 macaques were allowed to progress to endpoint criteria. Control group macaques were euthanized on Day 6.

#### 2.2.1. Physical Examinations

**Figure 1:**
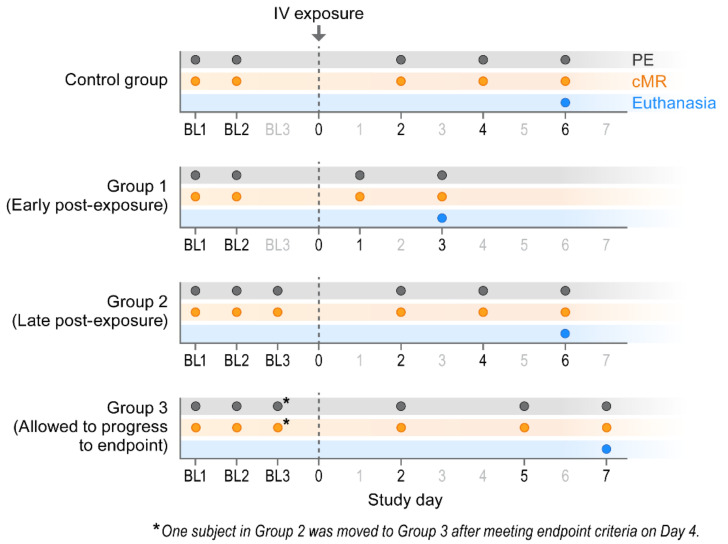
Experimental design. BL = baseline sampling, time points prior to intravenous (IV) cowpox virus (CPXV) exposure. PE = physical exam, cMR = cardiac magnetic resonance.

Macaques were exposed via the IV route to 6.7 log_10_ PFU of CPXV, observed daily for clinical signs of disease, and periodically anesthetized with ketamine for physical exams (Figure 1). Physical exams included blood withdrawals, weight measurements, and cMRI (Figure 1). Blood was drawn by venipuncture and collected in serum separator tubes or tripotassium ethylenediamine tetraacetic acid (K3 EDTA) tubes (BD Biosciences, Franklin Lakes, NJ, USA) for downstream assays. Complete blood count with differential analysis was performed using a Sysmex XS1000i automated system (Sysmex USA, Chicago, IL, USA). Serum chemistries were obtained on a COBAS INTEGRA 400Plus (Roche Diagnostics, Basel, Switzerland). The serological markers of myocardial necrosis and infarction—creatine kinase (CK), CK myocardial band (CK-MB), aspartate aminotransferase (AST), and lactate dehydrogenase (LDH)—were measured [9]. We must note that hemolysis may artifactually elevate CK, AST, and LDH values [10]. Commercial cardiac troponin assays were evaluated in-house for use on macaques, but the evaluated assays were associated with high background, suggesting poor cross-reactivity with macaque troponins. Furthermore, previous work indicated that 19% of crab-eating macaques had autoantibodies to troponin I, which likely interfered with measurement of circulating troponin I [11], supporting the high background observed and suggesting limited utility of cardiac troponins in evaluating cardiac damage in macaque models of disease.

#### 2.2.2. Clinical Endpoint

Group 1 macaques were euthanized on Day 3 post-exposure (Figure 1). Group 2 macaques were euthanized on Day 6, with the exception of one macaque that unexpectedly met clinical endpoint euthanasia criteria and was euthanized on Day 4. Group 3 macaques met clinical endpoint criteria on Day 7. Except where noted, the Group 2 macaque that met euthanasia criteria on Day 4 was included as a Group 3 macaque for data analysis. For Group 3 macaques, euthanasia criteria was based on scoring that included a combination of overall clinical appearance and signs of hemorrhagic disease, elevated respiratory rate, mucous membrane color, and dyspnea, responsiveness, recumbency, and hypothermia.

### 2.3. Virology, Histopathology, Immunohistochemistry (IHC), and Electron Microscopy

Complete necropsies were performed to collect tissues for virological and histopathological analyses. To determine infectious virus titers, tissues were homogenized in phosphate-buffered saline (PBS; #10010-023, Gibco, Waltham, MA, USA) to a final volume of 10 or 20%, depending on sample size, clarified by centrifugation, and plaqued on grivet (Haplorrhini: Cercopithecidae: *Chlorocebus aethiops* (Linnaeus, 1758)) kidney epithelial (Vero E6) cells (#CRL-1586, ATCC, Manassas, VA, USA) as previously described [8].

For histopathology, tissues were immersion-fixed in 10% phosphate-buffered formalin for a minimum of 72 h. Samples were paraffin-embedded and sectioned to approximately 4 microns by a microtome. Fixed slides were then stained with hematoxylin and eosin (H&E) and examined by light microscopy. IHC assays were performed on a Bond Immunostainer: Briefly, tissue sections were deparaffinized and rehydrated; heat-induced antigen retrieval was performed using citrate (pH 6.0) at 100 °C for 25 min. Samples from previous in which nonhuman primates were exposed to monkeypox virus and known to have high reactivity were used for positive controls. Tissues from normal macaques served as negative controls. Orthopoxvirus antigen was identified via IHC using a biotinylated rabbit anti-vaccinia virus polyclonal antibody (1:2000; Catalog #YVS8101, Accurate Chemical, Westbury, NY, USA), incubated for 15 min at room temperature. Primary antibody was localized with horseradish peroxidase and diaminobenzidine substrate. For negative controls, buffer was used in place of the primary antibody. Heart pathology scoring was based on severity of hemorrhage, necrosis, and inflammation. All histopathology samples were viewed and scored by a board-certified veterinary pathologist who was blinded to animal and group information. Scores were based on the percentage of area affected: 0 indicated normal tissue; 1 indicated less than 1% of area affected; 2 indicated 1–10%; 3 indicated 11–25%; 4 indicated 26–50%; 5 indicated more than 50% [12,13].

For conventional thin-section microscopic evaluation, excised tissues were preserved and inactivated for 72 h with 2.5% glutaraldehyde, 2.0% paraformaldehyde (E.M. Sciences, Warrington, PA, USA) in Millonig’s sodium phosphate buffer (Tousimis Research, Rockville, MD, USA). After inactivation was complete, tissues were washed repeatedly in Millonig’s buffer and incubated for 2 h in 1.0% osmium tetroxide (E.M. Sciences), in the same buffer. Following rinsing steps in ultrapure water and en bloc staining with 2.0% uranyl acetate, samples were dehydrated in a series of graded ethanols, infiltrated, and embedded in Spurr’s plastic resin (E.M. Sciences). Embedded blocks were sectioned using an Ultramicrotome Leica EM UC7 (Leica, Wetzlar, Germany). Sections with a thickness of 70–80-nm were collected on 200-mesh copper grids and stained with Reynolds lead citrate solution. Samples were examined in a Tecnai Spirit transmission electron microscope (FEI, Hillsboro, OR, USA), operating at 80 kV.

### 2.4. Whole Blood Flow Cytometry

Whole blood samples were collected from macaques, and a volume of 50 µL was assayed using BD TruCount tubes (BD Biosciences). Fluorescent reagents included protein tyrosine phosphatase receptor type C (PTPRC, formerly CD45) PerCP (#558411/D058-1283, BD Biosciences), CD3 Alexa Fluor 700 (#557917/SP34-2, BD Biosciences;), CD4 Alexa Fluor 488 (#317420/OKT4, Biolegend, San Diego CA, USA), CD8 antigen-presenting cell (APC) H7 (#641400/SK1, BD Biosciences), CD14 Pacific Blue (#558121/M5E2, BD Biosciences), Fc fragment of immunoglobulin G (IgG) receptor IIIa (FCGR3, formerly CD16) APCs (#561248/3G8, BD Biosciences), membrane spanning four domains A1 (MS4A1, formerly CD20) phycoerythrin-cyanine dye (PE-Cy7; #335793/L27, BD Biosciences), and killer cell lectin-like receptor C1 (KLRC1/natural killer cell receptor 2A [NKG2A]/formerly CD159a) phycoerythrin (PE) (#IM3291U/Z199, Beckman Coulter, Pasadena, CA, USA). Whole blood was incubated with the antibody mixture in a total staining volume of 100 µL for at least 20 min and fixed with 1X FACS lysing buffer (#349202, BD Biosciences) for at least 30 min. All incubations were performed at room temperature. Data acquisition and analyses were performed using a BD LSR Fortessa SORP Flow Cytometer using BD FACSDiva software (BD Biosciences). Fluorescent compensation was performed using IgG1κ CompBeads (#552843, BD Biosciences), stained with antibody for at least 20 min, washed with PBS, and fixed with FACS lysing buffer for at least 30 min.

### 2.5. Cardiac Sample Flow Cytometry

Heart samples were weighed and processed to single-cell suspensions. Collagenase type II (#17101-015, Gibco) was dissolved to 24,000 M and l U/mL in Hanks’ balanced salt solution (HBSS; #221-023-CV, Cellgro Mediatech, Corning, NY, USA), and DNase I (#10-104-159-009, Roche;) was dissolved to 4000 M and l U/mL in HBSS containing calcium (Ca^2+^) and magnesium (Mg^2+^) ions [12-022-CV, Cellgro Mediatech, Corning]. The collagenase solution was filter-sterilized through a 0.22-µm filter and aliquots were frozen. At the time of sample processing, fresh working stocks of DNase I (100 U/mL) and collagenase type II (600 U/mL) were prepared, using advanced Roswell Park Memorial Institute medium (RPMI) 1640 medium (#12633-01, Gibco), supplemented with 10% heat-inactivated fetal bovine serum (FBS; #F2442, Sigma-Aldrich). Heart sections were minced with scissors in the collagenase/DNase working stock and incubated for 15–30 min at 37 °C. Tissues were ground in a 100-µm cell strainer using the back end of a sterile plunger from a 5-mL syringe. Samples were passed through a 40-µm cell strainer, washed in RPMI 1640 medium, and supplemented with 10% heat-inactivated FBS (RPMI-10), followed by lysing with ammonium-chloride-potassium (ACK; #118 156 101, Quality Biological, Gaithersburg, MD, USA) to remove red blood cells. Samples were washed again with RPMI-10 and resuspended in PBS supplemented with 2% heat-inactivated FBS. Cells were counted using an Invitrogen Countess Automated Cell Counter (Life Technologies, Carlsbad, CA, USA) and assayed with BD TruCount tubes via the same method used for whole blood.

### 2.6. Cytokine Measurements

Cytokine concentrations were measured using a custom Milliplex kit (Millipore, Danvers, MA, USA) developed for nonhuman primates. We targeted the C–C motif chemokine ligand 2 (CCL2), a chemoattractant for recruiting monocytes, memory T cells, and dendritic cells to infection sites [14]; C-X-C motif ligand 8 (CXCL8), a chemoattractant for neutrophils that plays an important role in neutrophil activation and degranulation in a localized immune response [15]; and interleukin 6 (IL-6), a pleiotropic cytokine that is important in host defense and regulation of the inflammatory response and that mediates fever and acute phase response [16]—all of which are associated with endothelial leakage [17]. Briefly, a volume of 50 µL of macaque plasma was assayed in duplicate with magnetic beads, conjugated to antibodies of the desired analyte. After overnight incubation and agitation at 4 °C, plates were washed, anti-human detection antibodies were added, and streptavidin-phycoerythrin was added as a fluorescent detector. Following incubation with these components, plates were washed, the beads were resuspended in sheath fluid (Luminex, Austin, TX, USA), and beads in individual wells were detected using the FlexMap3D system (Luminex). Sample values were computed by a 4-parameter logistic regression against a standard curve of analytes provided with the kit. The lower limit of quantification (LLOQ) and upper limit of quantification (ULOQ) for analytes were: 38 and 10,477 pg/mL for CCL2; 10 and 12,339 pg/mL for CXCL8; 11 and 10,000 pg/mL for IL-6, respectively.

### 2.7. Cardiac Magnetic Resonance Image Acquisition and Data Analysis

cMRI procedures were performed while macaques were anesthetized and intubated to ensure compliance with breath-hold requirements. MRIs were performed using an Achieva 3.0T scanner (Philips North America, Andover, MA, USA), constructed to operate in a BSL-4 vivarium [18], with software v3.2.1.0 and Philips Flex SENSitivity Encoding (SENSE) radiofrequency (RF) receiver coils (Philips North America, Andover MA, USA). Multiple two-dimensional cine RF-spoiled gradient-echo images were acquired in both short and long axes. Cine images were used to assess ejection fraction as a surrogate measurement of cardiac function. A single-slice multi-echo fast field echo (FFE) acquisition was used to acquire T2* measurements, which were used to assess areas of perivascular micro-hemorrhage. Single-slice acquisition was necessary to minimize breath hold requirements for the anesthetized macaques. Slices were prescribed over anatomy with suspicious motion or edema on cine images. Data are presented here with all baselines averaged to establish a mean and one standard deviation left ventricular ejection fraction (the proportion of blood that is ejected from the heart during systole measured as the percent reduction in the blood volume from diastole [19]) normal range for the cohort on the available MR scanner.

MIM 5 software (MIM Software, Beachwood OH, USA) was used for data analysis of cine images. To measure cardiac ejection fraction, left ventricular volume was measured at systole and diastole. A supervised region growing segmentation algorithm was utilized with a lower threshold set to 2.5 standard deviations below the average ventricular signal intensity. MATLAB (Mathworks, Natick MA, USA) was used to create T2* maps from the multi-echo FFE acquisitions. Two-dimensional regions of interest were drawn using MIM software to measure myocardial T2* values since they can provide a characterization of cardiac hemorrhagic lesions [20,21].

## 3. Results

To study CPXV-induced cardiac lesions, a natural history experiment utilizing three groups of three crab-eating macaques were each exposed to 6.7 log_10_ PFU of CPXV strain Brighton Red. Group 1 consisted of two males and one female (age range 7–9 yr); Group 2 consisted of two males and one female (age 7 yr); Group 3 consisted of two males and one female (age range 7–10 yr); and the control group consisted of one male and two females (age range 7–10 yr).

**Figure 2:**
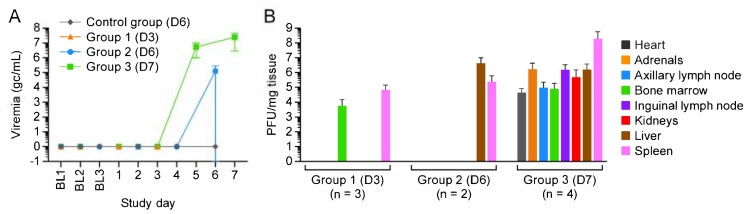
Viremia and tissue virus loads indicate early infection of the bone marrow and spleen. (**A**) Viremia was observed at Day 5 post-exposure. (**B**) Tissue viral load suggests early infection of the spleen and bone marrow. BL = baseline sampling, time points prior to intravenous (IV) cowpox virus (CPXV) exposure. Bars indicate standard deviation. Day 0 is not included on the graphs because no measurements were made.

**Figure 3:**
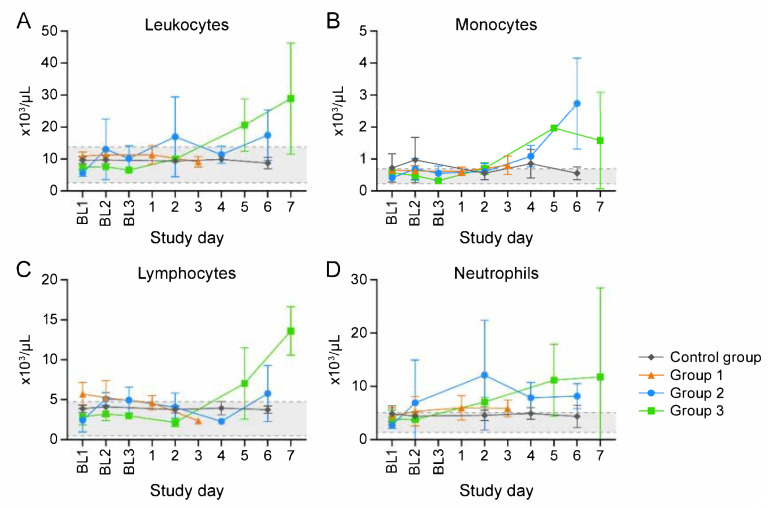
Clinical pathology supports inflammatory response. Total leukocyte (**A**), monocyte (**B**), lymphocyte (**C**), and neutrophil (**D**) number changes indicate inflammation. BL = baseline sampling, time points prior to intravenous (IV) cowpox virus (CPXV) exposure. Error bars reperesent standard deviation. Day 0 is not included on the graphs because no measurements were made.

Macaques in Group 1, Group 2, and Group 3 were euthanized 3, 6, and 7 d post-exposure (Day 3, Day 6, and Day 7), respectively. (One Group 2 macaque unexpectedly met euthanasia criteria on Day 4.) Three control group macaques, which were not exposed to virus, were euthanized on Day 6 (Figure 1). CPXV peripheral blood and tissue titers (Figure 2) and complete blood counts with differential analysis (Figure 3) reflected and built upon the results of our previous study [8]. Overall, the total leukocyte count increased from baseline, possibly due to systemic increases in pro-inflammatory cytokine concentrations.

**Figure 4:**
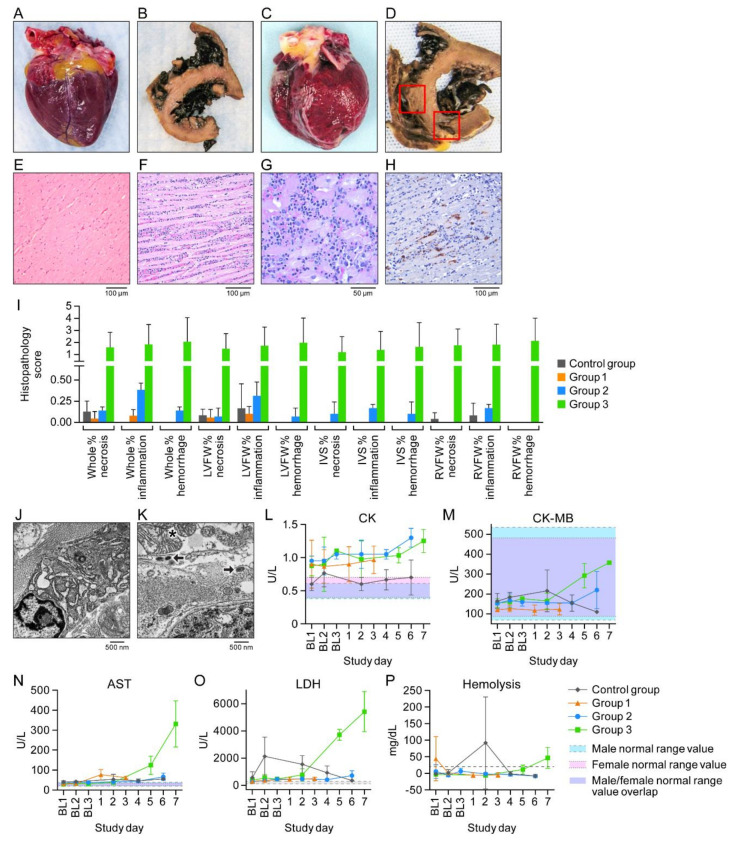
Cowpox virus (CPXV) directly infects cardiac tissue. (**A**,**B**) Gross pathology image of a fresh, normal heart and formalin-fixed and transversely sectioned normal heart tissue (control group macaques). (**C**,**D**) Petechiae and areas of hemorrhage and necrosis (Group 3 macaques). (**E**,**F**) Normal control group and Group 3 ventricular myocardium section with florid inflammation. (**G**) Higher magnification of F showing intracytoplasmic inclusion bodies and inflammatory infiltrate. (**H**) CPXV antigen (brown) in cardiac fibroblasts. (**I**) Histopathology scoring. (**J**,**K**) Normal control group and Group 3 myocardium. Asterisk: mitochondrial crystolysis; arrows: CPXV particles in cardiac fibroblast. (**L**–**P**) Creatine kinase (CK), CK myocardial band (CK MB), aspartate aminotransferase (AST), lactate dehydrogenase (LDH), and hemolysis over time. BL = baseline sampling, time points prior to intravenous (IV) CPXV exposure. Day 0 is not included on the graphs because no measurements were made.

Importantly, in contrast to the macaques of other groups, all Group 3 macaques had detectable infectious virus in the heart (Figure 2). Heart tissue was grossly normal in control group, Group 1, and Group 2 animals (Figure 4A,B); however, for Group 3 animals, petechia were observed on the pericardial surfaces, and myocardial damage and hemorrhage were observed (Figure 4C,D).

Histologic and transmission electron microscope analysis showed normal myocardium in control group animals (Figure 4E) but extensive lymphohistiocytic infiltrates with inclusion bodies (Figure 4F,G) in Group 3 animals. Histopathology scoring supported moderate myocarditis in Group 3 subjects (Figure 4J). CPXV antigen (Figure 4H), CPXV particles within cardiac fibroblasts, and mitochondria with crystolysis (Figure 4J,K) were present. Cardiac involvement was reflected by elevated CK, CK-MB, AST, and LDH activities (Figure 4L–P), although there was mild hemolysis (Figure 4P).

**Figure 5:**
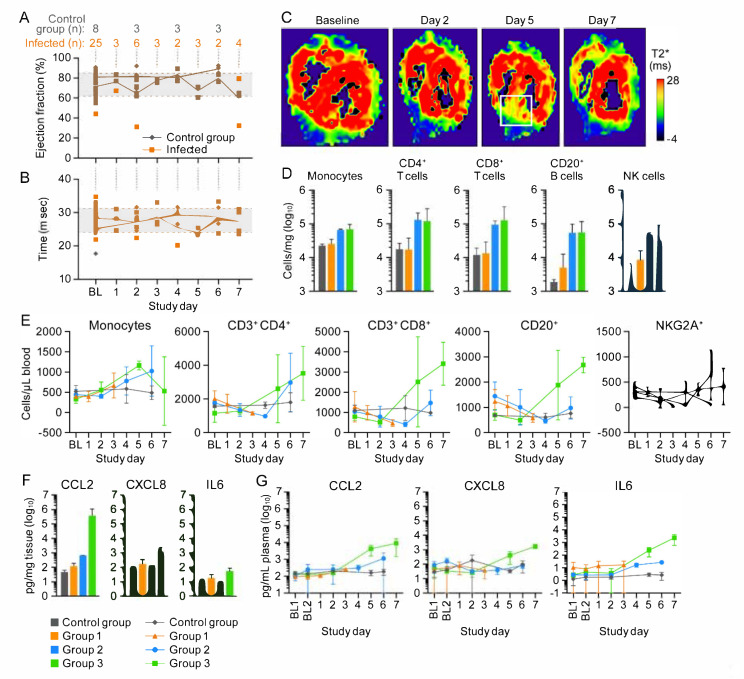
Myocardial hemorrhage and lymphohistiocytic inflammation in cowpox-virus-exposed macaques does not severely impact heart function. (Baseline values were averaged to establish a mean and one standard deviation left ventricular ejection fraction [LVEF] normal range.) (**A**) Changes in LVEF. (**B**) Myocardial hemorrhage as measured by T2*. (**C**) T2* maps of the most severely affected macaque. White square: area with increased T2*, indicating hemorrhage. (**D**) Peripheral blood mononuclear cells (PBMCs) infiltrated the heart tissue and peak at Day 6 post-exposure. (**E**) Circulating PBMC numbers increased by Day 4. Monocyte numbers declined in circulation by Day 7 but remained constant within the myocardium. (**F**,**G**) Heart tissue and plasma cytokine concentrations increased as disease progressed. Day 0 is not included on the graphs because no measurements were made.

All macaques underwent in vivo cMRI to quantitatively assess cardiac function during CPXV-induced disease. The left ventricular ejection fraction, chosen to assess cardiac function, was macaque-dependent and variable within groups but did not exceed the baseline range (Figure 5A). Likewise, T2*, a measure of myocardial hemorrhage, did not appreciably deviate from baseline values in any group (Figure 5B). As expected, when analyzed as individuals, T2* decreased at Day 5 and when subjects met clinical endpoint data suggested that Group 3 macaques developed increased hemorrhages compared to the other macaques. T2* maps identified regions of possible hemorrhage (Figure 5C) on Day 5. Together, these data indicate that, despite CPXV infection and inflammation of the myocardium, cardiac function was not adversely affected.

Cardiac tissue was immunophenotyped to characterize tissue response to infection. Group 2 and Group 3 macaques had higher absolute numbers of CD14^+^ monocytes, CD4^+^ T cells, CD8^+^ T cells, MS4A1^+^ B cells, and natural killer cells compared to Group 1 and control group macaques (Figure 5D). Because the heart is highly vascularized, circulating blood samples were also immunophenotyped and assessed through complete blood counts with differential analysis. Absolute numbers of these cells in whole blood (Figure 5E) indicated their mobilization from bone marrow, proliferation in circulation, or both as well as monocyte homing to the myocardium.

Plasma and myocardial cytokine concentrations were analyzed during the course of CPXV infection to determine whether myocardial tissues had increased CCL2, CXCL8, and IL-6, which are associated with endothelial leakage [17]. CCL2, CXCL8, and IL-6 concentrations were elevated in response to CPXV infection:

CCL2 belongs to the CC chemokine family and is a chemoattractant for recruiting monocytes, memory T cells, and dendritic cells to infection sites [14]. CCL2 concentrations observed in the heart and plasma in CPXV infection are consistent with the recruitment of monocytes into the myocardium. CCL2 concentrations (Figure 5F) in the myocardium of Group 3 macaques were 503,779 pg/mg, which is 3.9 log_10_ higher than the value measured in control group macaques and 575-fold higher than that measured in Group 2 macaques. CCL2 concentrations in the heart were compared to CCL2 in circulation within the control group macaques at Day 6 (Figure 5G). We found a 0.25-fold difference between heart tissue and circulating blood, suggesting that, under normal conditions, the heart produces little CCL2. Circulating CCL2 (Figure 5G) in control group macaques increased 1.2-fold between baseline and Day 6 (156.2 and 186.8 pg/µL, respectively), suggesting little impact on cytokine dynamics from the repeated procedures and extended anesthesia required for cMRI. By comparison, CCL2 concentration in circulation increased 48-fold between Group 3 macaques and the control group macaques (8987.0 and 186.8 pg/µL, respectively) at their respective terminal days. The CCL2 concentration was eight-fold higher between Group 2 on Day 6 (1137.97 pg/µL) and Group 3 at terminal endpoint (Day 7, 8987.0 pg/µL). A two-fold increase was observed between Day 5 (4296.33 pg/µL) and Day 7.

CXCL8 chemoattracts neutrophils and plays an important role in neutrophil activation and degranulation in a localized immune response [15]. The CXCL8 concentration (Figure 5F) in the myocardium increased 15-fold at Day 7, (1315.64 pg/µL) compared to control group macaques (86.33 pg/µL) at Day 6. The CXCL8 myocardial concentration was 78-fold higher in Group 3 (1315.64 pg/µL) than in Group 1 (168.95 pg/µL) and 10-fold higher than in Group 2 (128.93 pg/µL) macaques at terminal time point. Circulating CXCL8 concentrations (Figure 5G) among CPXV-exposed macaques increased from Day 5 to Day 7 by 5.6-fold within Group 3. At terminal blood time point, Group 3 macaques had 18-fold higher CXCL8 concentrations (1421.71 pg/µL) than the control group (Day 6; 73.11 pg/µL), 17-fold higher than Group 2 (Day 6; 79.37 pg/µL), and 36-fold higher than Group 1 macaques (Day 3; 38.00 pg/µL).

IL-6 is a pleiotropic cytokine that is important in host defense and regulation of the inflammatory response, and it mediates fever and acute phase response [16]. IL-6 concentrations in the hearts followed a similar pattern as CCL2 and CXCL8, but increases in concentrations were lesser in magnitude. The terminal myocardial concentrations (Figure 5F) of IL-6 in Group 3 macaques was 5.6-fold higher than the control group on Day 6 (55.05 pg/µL and 9.76 pg/µL, respectively), six-fold higher than Group 2 macaques on Day 6 (9.16 pg/µL), and three-fold higher than Group 1 macaques (17.71 pg/µL). Circulating IL-6 concentrations increased steadily as disease progressed (Figure 5G). Group 3 macaques had an 87.9-fold increase in IL-6 concentrations compared to control group macaques (2351.61 pg/µL and 2.67 pg/µL, respectively) at terminal time point—82-fold higher than Group 2 macaques (26.84 pg/µL), and 138-fold higher than Group 1 macaques (16.99 pg/µL). A 15-fold increase was observed between Day 5 and Day 7 among Group 3 macaques (152.55 pg/µL for Group 3 at Day 5). These data indicated that the myocardium is undergoing an inflammatory response due to direct infection of cardiac fibroblasts that results in hemorrhage into the myocardium.

## 4. Discussion

Human hemorrhagic smallpox was characterized as a “toxemia” with reports of myocardial and subendocardial hemorrhage [5,7,22,23]. The data demonstrated that myocarditis was acute and occurred late in disease, which suggests that the myocardial and subendocardial hemorrhage observed in humans was also a near-terminal event. Inflammatory myocarditis can result in lowered cardiac output and decreased tissue perfusion, possibly contributing to distributive shock [24]. Unfortunately, the technological advances used in this experiment were not available when VARV was circulating. Therefore, direct comparison of cMRI, cytokine analysis, and flow cytometry data to human case reports cannot be performed. However, animal model data can help guide treatments to similar diseases. In this study, serial sampling was performed to characterize cardiac response to CPXV infection, and the data suggest a pro-inflammatory response that leads to an acute myocarditis but no changes in cardiac output.

In our model, like human disease, the development of myocarditis was acute and occurred at end stages of disease. Examination of the heart at early, late, and terminal time points indicated that myocarditis was acute, with most changes observed during Day 6 and Day 7. Histology and ultrastructural analyses demonstrated hemorrhage, necrosis, and inflammation of the myocardium at Day 7. cMRI indicated that LVEF was not diminished during disease course. However, the data must be considered suggestive because high variability within and between groups was observed. As an adaptation to the rapid heart rate of crab-eating macaques, cMRI was performed on a single axial slice; thus, areas of hemorrhage may have been missed due to sampling inefficiency.

Interestingly, the T2* cMRI suggested mild hemorrhage into the myocardium, which was corroborated by gross pathology and histopathology as well as changes in CK-MB, CK, AST, and LDH (which occurred from Day 5 through terminal endpoint in CPXV-exposed macaques). CK-MB, CK, AST, and LDH were chosen as analytes to evaluate cardiac disease because previous work indicated that 19% of crab-eating macaques had autoantibodies to troponin I [11].

Inflammation, exudation, and necrosis within the myocardium may also contribute to the development of arrhythmias, during either the acute or chronic (reparative) phase, with increased potential for sudden cardiac death [25,26,27]. Such arrhythmias generally have no clearly discernible structural correlate that can be identified histologically and are diagnosed only by electrocardiogram (EKG) [28]. Additionally, specific systematic sampling of the cardiac conducting system (atrioventricular [AV] node, sinoatrial [SA] node, and bundle branches) for histology was not performed, thus arrhythmogenic lesions to these tissues may have been missed. Future studies incorporating continuous telemetry monitoring of cardiac rhythm and systematic histologic evaluation of the conduction system in this model may be informative.

Establishing direct infection of the myocardium and its associated inflammatory response and characterizing the changes in cardiac output provide insight into pathogenesis. In this study, macaques developed cardiac lymphohistiocytic infiltrate with hemorrhage and cells positive for CPXV antigen. Also, positive cardiac fibroblasts were prominently observed via electron microscopy in the Group 3 macaques that met endpoint criteria at Day 7—but not in Group 2 macaques, which were euthanized on Day 6. Immunological analysis showed increased CCL2 concentration and increased absolute numbers of CD14^+^ monocytes in the myocardium, suggesting that pro-inflammatory CCL2 production recruited monocytes into the infected heart tissue. The increased absolute numbers of CD14^+^ monocytes in the myocardium of Group 3 macaques compared to the control group and Group 2 supports a rapid accumulation of cells. Furthermore, the decline in absolute numbers of monocytes in circulation was not observed among the other assayed leukocyte populations.

The decline in circulating monocytes with concomitant increase in total white blood cell numbers, increased tissue monocyte concentration, increased CCL2 concentration in the myocardium, and rapid onset of bone marrow hypocellularity suggest that CD14^+^ monocytes were being recruited from the circulation into the tissue. Experimental limitations prevented both bone marrow and peripheral blood mononuclear cell (PBMC) infiltrate analysis in other tissues to determine whether this effect was systemic or localized. High CCL2, CXCL8, and IL6 concentrations are frequently observed in septic shock [17] and have been associated with endothelial cell contraction, which could lead to coagulopathy and hemorrhage [29]. Together, these data suggest that CCL2 production is increased in the heart and results in attracting macrophages to the heart, which may further drive disease. CCL2, CXCL8, and IL6 may be correlative biomarkers of CPXV-mediated hemorrhagic manifestations of the disease. Thus, therapies targeting these cytokines may prevent endothelial leakage, which would reduce dehydration and hemorrhage. As such, the CPXV infection model of hemorrhagic smallpox provides an opportunity to evaluate the effectiveness of immunomodulators targeting CCL2 and other pro-inflammatory mediators during progression of viral-induced hemorrhagic diseases.

Serum chemistry analysis and complete blood count with differential data suggest rapid disease onset with an increase in lymphocytes, monocytes, and neutrophils numbers (beginning Day 3 and continuing increase to endpoint), possibly due to systemic increases in pro-inflammatory cytokine concentrations [30]. These data also coincided with increases in circulating CCL2, CXCL8, and IL6 concentrations. Typical for viral infections, CPXV likely stimulated cytokine production by activating macrophages or monocytes, which led to the downstream pro-inflammatory response. As infection progressed, the pro-inflammatory state increased, likely due to active suppression of the immune response, which is common to orthopoxviruses [31,32]. Unlike other infection routes or virus infection of non-myeloid lineage target cell types, infection of monocytes or macrophages cells likely leads to a dysregulation of cytokine production, which further stimulates a pro-inflammatory response that is ultimately harmful to the host. A similar scenario is observed for filovirus infection of macrophages [33].

## 5. Conclusions

The data generated in this experiment support rapid onset of a mild myocarditis that minimally impacted cardiac function, though the rapid course of disease may have prevented observation of myocarditis leading to decreased cardiac function. Nonetheless, these study data have provided further characterization of CPXV-induced cardiac disease in the crab-eating macaque CPXV human hemorrhagic smallpox model. Furthermore, application of cMRI to other models of virus-induced hemorrhagic disease—such as, the nonhuman primate models for Ebola virus and Marburg virus diseases or mammarenavirus infections—may provide enhanced insight into the disease mechanisms and possible broadly applicable treatments for viral hemorrhagic diseases.

## Data Availability

Data will be made available on request.
